# Non-histone lactylation: unveiling its functional significance

**DOI:** 10.3389/fcell.2025.1535611

**Published:** 2025-01-24

**Authors:** Pusong Shi, Yongjie Ma, Shaolu Zhang

**Affiliations:** ^1^ School of Integrative Medicine, Tianjin University of Traditional Chinese Medicine, Tianjin, China; ^2^ Laboratory of Cancer Cell Biology, Tianjin Medical University Cancer Institute and Hospital, National Clinical Research Center for Cancer, Tianjin, China; ^3^ Tianjin’s Clinical Research Center for Cancer, Tianjin, China; ^4^ Key Laboratory of Breast Cancer Prevention and Therapy, Ministry of Education, Tianjin Medical University, Tianjin, China; ^5^ Key Laboratory of Cancer Prevention and Therapy, Tianjin, China; ^6^ State Key Laboratory of Druggability Evaluation and Systematic Translational Medicine, Tianjin, China

**Keywords:** non-histone, lactylation, posttranslational modification, lysine, metabolism

## Abstract

Lactylation, a newly discovered protein posttranslational modification (PTM) in 2019, primarily occurs on lysine residues. Lactylation of histones was initially identified, and subsequent studies have increasingly demonstrated its widespread presence on non-histone proteins. Recently, high-throughput proteomics studies have identified a large number of lactylated proteins and sites, revealing their global regulatory role in disease development. Notably, this modification is catalyzed by lactyltransferase and reversed by delactylase, with numerous new enzymes, such as AARS1/2, reported to be involved. Specifically, these studies have revealed how lactylation exerts its influence through alterations in protein spatial conformation, molecular interactions, enzyme activity and subcellular localization. Indeed, lactylation is implicated in various physiological and pathological processes, including tumor development, cardiovascular and cerebrovascular diseases, immune cell activation and psychiatric disorders. This review provides the latest advancements in research on the regulatory roles of non-histone protein lactylation, highlighting its crucial scientific importance for future studies.

## 1 Introduction

Metabolic dysregulation is one of the most distinctive characteristics of cancer cells, with the famous Warburg effect being a classical example of such metabolic changes (Icard et al., 2018; Long et al., 2024). This effect reveals that cancer cells, even in the presence of sufficient oxygen, still produce large amounts of lactate. Furthermore, numerous studies have shown that many physiological and pathological processes, especially cancer development, rely mainly on glycolysis and lactate production. As an intermediate metabolite, lactate plays a pivotal role in promoting tumor cell proliferation, metastasis, and immune suppression. Investigations into lactylation have revealed their crucial functions in both physiological and pathological processes (Zhang et al., 2019a; Zhang et al., 2019b; Daw et al., 2020). However, owing to limitations in detection technology, early studies on lactylation focus primarily on its epigenetic role in histone lactylation, which influences histone modifications, chromatin remodeling, and transcriptional regulation (Yang et al., 2023a; Li et al., 2024a; Li et al., 2024e; Wang et al., 2024a). Findings from proteomic studies have revealed that, in addition to histones, a significant number of non-histone proteins undergo lactylation. Unlike histone lactylation, which modifies H3 and H4 primarily via histone deacetylases (HDACs) and histone acetyltransferases (HATs), non-histone proteins impact chromatin structure and function by influencing a range of non-histone components, including metabolic enzymes, transcription regulatory factors, and signal transduction molecules (Jiang et al., 2024b). Preliminary evidence has confirmed that lactylation on non-histone substrates in humans also plays a critical regulatory role (Xin et al., 2022; Yang et al., 2023b; Wang et al., 2024b). Over the past period, research on non-histone lactylation rapidly emerged. A total of 273 lysine lactylation (Kla) sites within 166 proteins, including 12 linked to fungal pathogenicity, were identified in *Botrytis cinerea* through a global lysine lactylome analysis performed by LC‒MS/MS (Gao et al., 2020). Among these proteins, the most notable one is mitogen-activated protein kinase (MAPK), which was identified as being lactylated at the K60 site, and citrate synthase, a rate-limiting enzyme in the tricarboxylic acid (TCA) cycle, which was also found to undergo lactylation at the K401 site, etc. Through the synthesis and analysis of model lactylated peptides, it was found for the first time that peptides carrying lactylated lysines form chain-like immonium ions during secondary fragmentation in the mass spectrometer collision chamber. These ions underwent deamination and cyclization, producing secondary fragments known as cyclic immonium ions (Wan et al., 2022). The findings from these chemical probes for lactylation confirmed the prevalence of lactylation. Therefore, this review summarizes the development and detection of non-histone lactylation, the transferases mediating these modifications, and the role of non-histone lactylation in pathological processes and potential therapies.

### 1.1 The metabolic process of lactate

Glucose primarily generates energy via two metabolic pathways: glycolysis and oxidative phosphorylation. After glycolysis, each glucose molecule produces two pyruvate molecules. In the presence of oxygen, pyruvate is converted to acetyl-CoA by pyruvate dehydrogenase (PDH) to enter the TCA cycle. However, in the absence of oxygen, pyruvate is converted to lactate by lactate dehydrogenase (LDH). This conversion of pyruvate to lactate can also take place under aerobic conditions—a phenomenon known as the “Warburg effect” (Brooks, 2020). According to the lactate shuttle hypothesis, lactate serves as a key messenger for delivering oxidative and gluconeogenic substrates and plays a role in cell signaling (Brooks, 2002). As a hydroxy carboxylic acid generated during glycolysis, lactate exists in two stereoisomers, L-lactate and D-lactate, with L-lactate being the predominant form produced during anaerobic glycolysis (Manosalva et al., 2021). Lactate is not merely a waste product; it can also be transported to other tissues for energy production. In the liver, lactate can be converted back to pyruvate by LDH and then used in gluconeogenesis to synthesize glucose (Ling et al., 2012). This process is part of the Cori cycle, in which lactate is transported from the muscles to the liver, converted to glucose, and then sent back to the muscles as an energy source. In neurons, lactate can be directly converted to pyruvate, which then enters the mitochondria for aerobic respiration to produce ATP (Genc et al., 2011).

**FIGURE 1:**
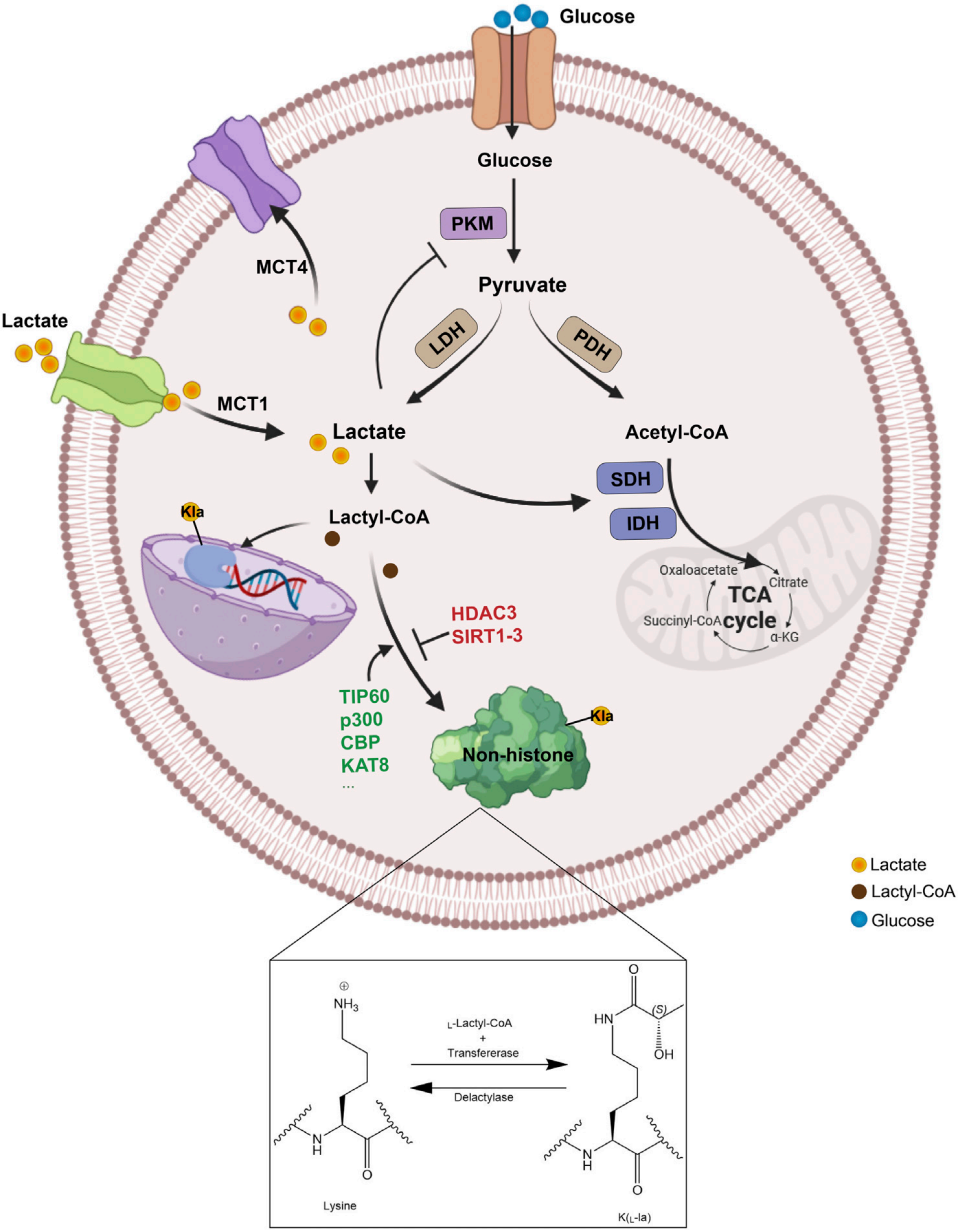
The metabolism and transport process of lactate. Lactate can be generated in multiple ways, produced intracellularly through glycolysis catalyzed by LDH or absorbed from the extracellular environment via MCT1, and then be converted to lactyl-CoA, which facilitates the lactylation of non-histone proteins by specific enzymes. Specifically, lysine lactylation occurs when the lactyl group is transferred to the lysine residue on a non-histone protein, with lactoyl-CoA serving as the donor and lactyltransferases catalyzing the transfer. Conversely, delactylases catalyze the reverse reaction, removing the lactyl group from the lysine residue to achieve lysine de-lactylation. PKM, glycolytic enzyme pyruvate kinase M; LDH, lactate dehydrogenase; PDH, pyruvate dehydrogenase; SDH, succinate dehydrogenase; IDH, isocitrate dehydrogenase; TCA, tricarboxylic acid; MCT, monocarboxylate transporter; Kla, lysine lactylation.

Lactate crosses the cell membrane via three mechanisms: free diffusion, anion exchange, and monocarboxylate transporters (MCTs), including MCT1 (SLC16A1), MCT2 (SLC16A7), MCT3 (SLC16A8), and MCT4 (SLC16A3), along with two sodium-coupled lactate cotransporters, SLC5A12 and SLC5A8 (Halestrap, 2012). In mammals, MCT1 and MCT4 are the main transporters involved in lactate transport. MCT1 is widely distributed and participates in lactate uptake in neutrophils and various organs, including the heart, skeletal muscle, red blood cells, and liver for gluconeogenesis. In contrast, MCT4, which is expressed in cells with high glycolytic activity, such as muscle fibers, increases its expression in response to hypoxia, facilitating the export of lactate from the cells to prevent intracellular acidosis (Halestrap and Meredith, 2004; Merezhinskaya et al., 2004; Ullah et al., 2006) (Figure 1).

### 1.2 Development and detection of assay techniques for lactylation

In histones and other proteins, lysine can undergo lactylation, forming a modification that links cellular metabolism to protein function. In recent years, post-translational modifications have been recognized to increase the complexity of protein function and regulation (Zhang et al., 2019a; Gaffney et al., 2020). Given the enhanced glycolysis and intracellular lactate accumulation in cancer cells, Kla plays a crucial role in cancers such as hepatocellular carcinoma (HCC) (Pan et al., 2022; Cheng et al., 2023; Wu et al., 2023; Yang et al., 2023b). In this context, the advancement of research techniques has made the identification of lactylation more precise and convenient.

In addition to conventional antibody-based detection methods, lactylation can also be discovered and detected via novel approaches. The development of chemical probes and ultrasensitive sensors for lactate metabolism provides a further technical guarantee for the study of lactylation (Li et al., 2023). With the continuous advancement of AI technology, recent progress in machine learning has enabled the development of computational models for Kla site prediction. This work not only enhances the understanding of Kla’s functional roles but also holds significant potential to drive future research in protein modification prediction and functional annotation (Guan et al., 2024).

Tao Peng’s team reported the development of a bioorthogonal chemical probe, YnLac, which enabled proteomic-level detection and identification of protein lactylation in mammalian cells for the first time (Sun et al., 2022). In this study, the researchers designed and synthesized the YnLac probe on the basis of the chemical structure of lactate. Researchers subsequently applied the probe for chemical proteomics analysis of lactylated substrate proteins in HEK293T cells. Using the probe and a pan-lactylation antibody, they confirmed lactylation signals in three selected substrate proteins, namely, high mobility group box 1 (HMGB1) (Yang et al., 2022), Nucleolin (NCL) (Yang et al., 2024b) and Poly (ADP-ribose) polymerase 1 (PARP1), demonstrating the reliability of the chemical proteomics results.

Hui Ye et al. first discovered that the cyclic immonium ion of lactyllysine, formed during tandem mass spectrometry, could be used as a marker to identify lactylation sites (Wan et al., 2022). The research team definitively established the sensitivity and specificity of cyclic immonium ions as reliable lactylation indicators after analyzing enriched, positive lactylated peptides from biological samples and using nearly 100,000 spectra of unmodified synthetic peptides as a negative control. With this diagnostic ion strategy, researchers revealed numerous novel lactylated substrate proteins and their modification site.

Notably, the Meltome Atlas data published in *Nature Methods* in 2020 (Jarzab et al., 2020), which explored proteome thermal stability in various human cell lines, revealed that lactylation was highly enriched in glycolytic pathway enzymes (Wang et al., 2023d). One of these enzymes, aldolase A (ALDOA), exhibited conserved lactylation across multiple human tumor cell lines with a high occupancy rate (Hu et al., 2024). Researchers have hypothesized that lactylation could regulate the activity of metabolic enzymes, thus mediating the glycolytic pathway. Lactylation at the K147 site of ALDOA revealed a new mechanism by which lactylation triggered the negative feedback loop of the glycolytic pathway, demonstrating how lactate accumulation led to downregulation of glycolytic activity through enzyme modification (Cheng et al., 2024).

### 1.3 Non-histone lactating modifications “writers” and “erasers”

Lactylation is a reversible and dynamic modification. Although several histone lactating modification enzymes such as E1A binding protein p300 (p300), sirtuin1-3 (SIRT1-3) and HDAC3 have been identified (Xie et al., 2022; Zhang and Zhang, 2024), the transferase mediating non-histone lactating modification has yet to be discovered (Yu et al., 2024a). Lactylation is a post-translational modification commonly mediated by lysine acetyltransferases that act as pan-Kla writers such as Tat-interactive protein 60 (TIP60), p300, Lysine acetyltransferase 8 (KAT8) and CREB-binding protein (CBP). Inhibiting lactylation through pharmacological blockade of p300 has been shown to significantly reduce neuronal death and attenuate glial activation following cerebral ischemia (Xiong et al., 2024). Furthermore, the suppression of CBP activity led to the downregulation of MRE11 lactylation, thereby impairing homologous recombination (HR) and increasing the chemosensitivity of tumor cells (Chen et al., 2024g). Notably, TIP60 has been identified as the lysine lactyltransferase for Nijmegen breakage syndrome 1 (NBS1), specifically facilitating the lactylation of NBS1 at K388 site (Chen et al., 2024b). In addition, Xie’s research reported that KAT8 has been identified as a pivotal global lactyltransferase, playing a crucial role in regulating eukaryotic elongation factor 1A2 (eEF1A2) lactylation. Furthermore, among the proteins that interacted with eEF1A2, the lactyltransferase KAT8 was capable of lactylating a variety of oncogenic proteins (Xie et al., 2024).

Recent studies revealed that, unlike commonly known acetylation-related writers, alanyl-tRNA synthetase 1 (AARS1) and alanyl-tRNA synthetase 2 (AARS2) are newly discovered lactylation writers. AARS1/2 exhibits strong binding affinity for L-lactate, catalyzing ATP-dependent lactylation on lysine residues (Ju et al., 2024; Li et al., 2024c). Zong et al. revealed that AARS1 functions as both a lactate sensor and a lactyltransferase, mediating global lysine lactylation. Specifically, AARS1-mediated lactylation of p53 impairs its liquid‒liquid phase separation, DNA binding, and transcriptional activation, thus contributing to tumorigenesis (Zong et al., 2024). In parallel, Mao et al. demonstrated that during mitochondrial protein lactylation, hypoxia induced the accumulation of AARS2, which facilitated the lactylation of lysine on pyruvate dehydrogenase alpha 1 (PDHA1) in the pyruvate dehydrogenase complex, as well as lysines on carnitine palmitoyltransferase 2 (CPT2) (Mao et al., 2024). This modification inactivates both enzymes and suppresses oxidative phosphorylation.

The common deacetylases HDAC1-3 (HDAC3 is the most effective “eraser” of both L- and D-lactate modifications) and SIRT1-3 also have the ability to delactylate (Dai et al., 2022a; Moreno-Yruela et al., 2022; Fan et al., 2023c). Chen reported that HDAC3 was the NBS1 delactylase (Chen et al., 2024b). SIRT1 and SIRT3 serve as pivotal “erasers” for Kla, revealing their selective modulation of both histone and non-histone proteins (Du et al., 2024; Hu et al., 2024). SIRT1-mediated delactylation could influence the cellular localization of Canopy 3 (CNPY3) to promote lysosomal rupture and trigger pyroptosis (Zhang et al., 2024b). On the other hand, when the lactylation of cyclin E2 (CCNE2) promoted the growth of HCC, SIRT3 induced apoptosis in HCC cells and inhibited the growth of HCC *in vivo* by regulating the Kla level of CCNE2 (Jin et al., 2023). The details of the writers and erasers of non-histone lactylation are provided in Table 1.

**TABLE 1:** Writers and Erasers of non-histone lactylation.

Category	Name	Target	Ref
Writer	TIP60	NBS1, Vps34	Jia et al. (2023), Chen et al. (2024b)
	p300	YY1, α-MHC, HMGB1, NCL, PKM2, Twist1, MeCP2, Snail1	Yang et al. (2022), Fan et al. (2023a), Wang et al. (2023a), Zhang et al. (2023a), Chen et al. (2024f), Huang et al. (2024a), Wang et al. (2024d), Xu et al. (2024), Yang et al. (2024b)
	CBP	HMGB1, MRE11, Twist1, Snail1	Yang et al. (2022), Fan et al. (2023a), Chen et al. (2024g), Xu et al. (2024)
	KAT8	eEF1A2, LTBP1	Xie et al. (2024), Zou et al. (2024)
	AARS1	p53, METTL16	Sun et al. (2023a), Zong et al. (2024)
	AARS2	PDHA1, CPT2, METTL16	Sun et al. (2023a), Mao et al. (2024)
	ATAT1	NAT10	Yan et al. (2024b)
Eraser	HDAC3	NBS1, MeCP2	Chen et al. (2024b), Chen et al. (2024f)
	SIRT1	CNPY3, α-MHC, PTBP1	Zhang et al. (2023a), Zhang et al. (2024b), Zhou et al. (2024b)
	SIRT2	METTL16	Sun et al. (2023a)
	SIRT3	CCNE2, PDHA1, CPT2	Jin et al. (2023), Mao et al. (2024)

## 2 The functional role of non-histone lactylation in cardiovascular diseases

**FIGURE 2:**
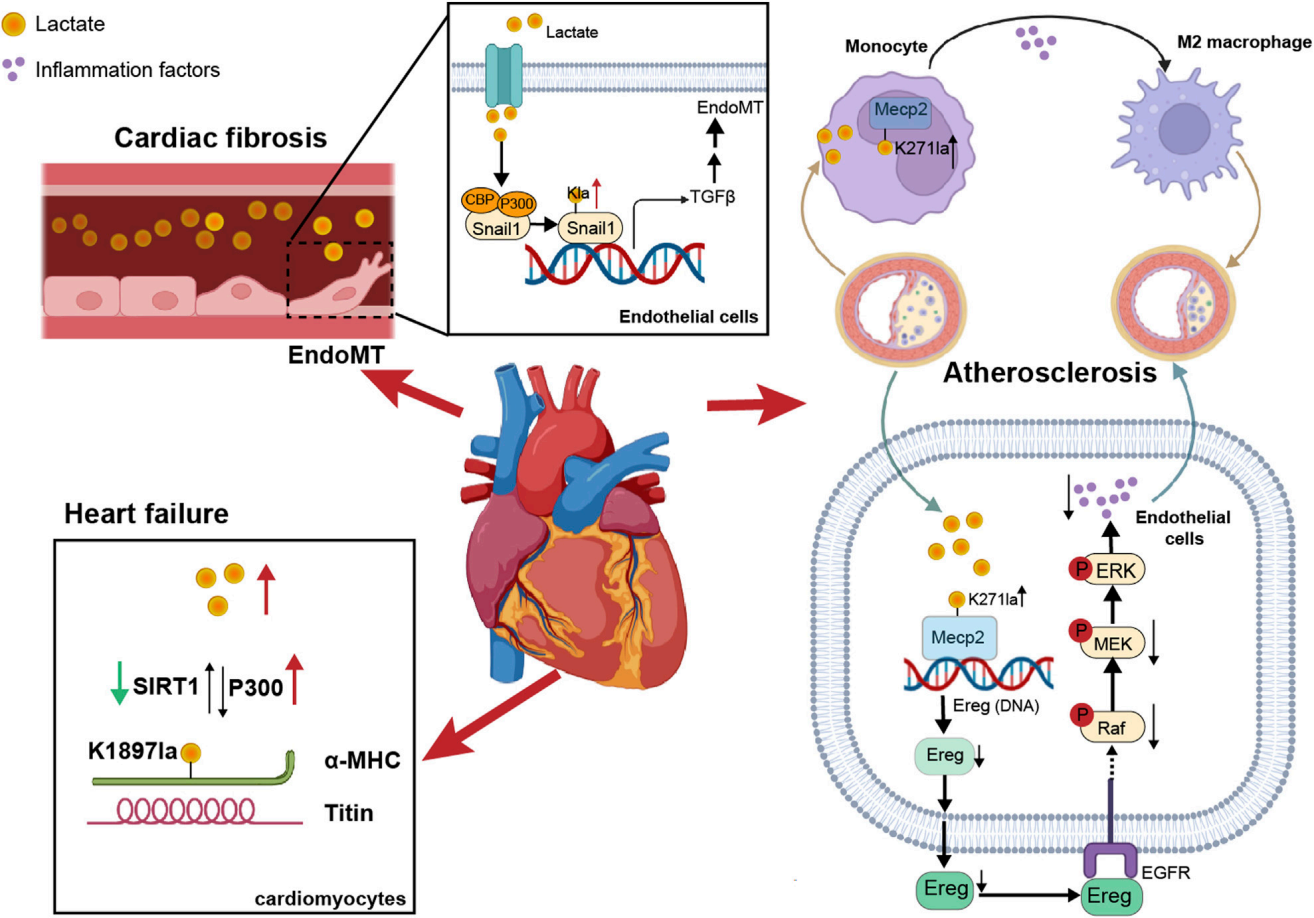
The impact of non-histone lactylation on cardiovascular diseases. On one hand, lactate in blood vessels induced lactylation in vascular endothelial cells, promoting EndoMT. On the other hand, lactylation of α-MHC in cardiomyocytes enhanced the binding between α-MHC and Titin, thereby reducing the risk of heart failure. Additionally, in atherosclerosis, the lactylation of Mecp2K271 promoted M2 macrophage polarization and inhibited inflammatory factors, thereby increasing plaque stability.

Cardiovascular disease (CVD) is a very common type of disease, with conditions such as acute coronary syndrome and heart failure (HF) being significant contributors to global mortality (Gandra et al., 2016; Ralapanawa and Sivakanesan, 2021). The occurrence and progression of CVD are closely tied to energy substrate metabolism, and alterations in endocrine function, inflammation, and hemodynamics can play pivotal roles in determining disease outcomes. As the concept of “cardiac rehabilitation” continues to gain traction in the realm of cardiovascular diseases (Brown et al., 2024; McEvoy et al., 2024), exercise plays a pivotal role in the prevention and treatment of CVDs. Lactate, a primary metabolite produced during exercise, has been shown in animal experiments and clinical studies to alleviate HF, inhibit inflammation, and delay myocardial hypertrophy when it is present at certain concentrations (Haege et al., 2021). Therefore, certain CVDs, such as HF and atherosclerotic cardiovascular disease (ASCVD), can benefit from exercise. However, other clinical studies have demonstrated that high levels of lactate are associated with an increased mortality rate among patients with HF (Zymliński et al., 2018; Biegus et al., 2019). Moreover, diseases such as acute myocardial infarction, HF, atrial fibrillation, and atherosclerosis often accompany accelerated glycolysis, leading to increased lactate production (Ouyang et al., 2023). Consequently, the underlying mechanisms still require further investigation. Insights into lactylation offer a new perspective for the prevention and treatment of CVDs (Figure 2).

Alpha-myosin heavy chain (α-MHC) and titin are essential for heart structure and contraction (Palmer, 2005; Linke, 2008). Zhang et al. reported that the lactylation of α-MHC at K1897 affected the global lactylation of α-MHC, which promoted a significant increase in the interaction between α-MHC and Titin and reduced the possibility of heart failure (Zhang et al., 2023a), and p300 and SIRT1 acted as lactyltransferase and delactylase enzymes of α-MHC, respectively. Moreover, the concentration of lactate in cardiomyocytes is also key to the lactylation of α-MHC. By intervening with sodium lactate or inhibiting the increase in the lactate concentration of the key lactate transporter MCT4 in cardiomyocytes, α-MHC K1897 lactylation and the α-MHC-Titin interaction can be promoted, which effectively alleviated heart failure caused by a decrease in α-MHC lactylation.

Myocardial infarction (MI) is a serious condition caused by ischemia and hypoxia of the myocardium. Lactate exacerbated cardiac dysfunction by promoting endothelial-to-mesenchymal transition (EndoMT) after MI, thereby increasing cardiac fibrosis. Lactate activation of the TGF-β/Smad2 pathway disrupted endothelial cell function and induced mesenchymal-like properties following hypoxia. Specifically, in response to hypoxia, lactate facilitated the nuclear translocation of Snail1 (a TGF-β transcription factor). Snail1 interacted with CBP/p300 (Li et al., 2020) to promote its lactylation through MCT-dependent lactate transporter signaling, ultimately inducing EndoMT (Fan et al., 2023a; Zhao et al., 2023).

It is known that exercise has a cardioprotective effect on ASCVD, which could enhance heart function and metabolism (Gibb and Hill, 2018). Chen’s team discovered that exercise facilitated the lactylation of methyl-CpG-binding protein 2 (MeCP2) at the K271 residue, which in turn promoted the polarization of M2 macrophages to exert an anti-inflammatory effect and enhanced plaque stability (Chen et al., 2024f). Moreover, exercise training promoted the lactylation of MeCP2 at the K271 residue in vascular endothelial cells. This process inhibited the transcription of epiregulin, subsequently suppressing the phosphorylation of epidermal growth factor receptor (EGFR) and the activity of MAPK. As a result, the progression of ASCVD was delayed (Wang et al., 2023b).

## 3 The functional role of non-histone lactylation in brain diseases

### 3.1 Effect of non-histone lactylation of neuroglial cells on craniocerebral disease

Neuroglial cells (NCs), which are abundant in the central nervous system (CNS), are classified into astrocytes, oligodendrocytes, and microglia (Xie et al., 2020). These cells play crucial roles in insulation, nutrition, support, and protection of neurons and are also associated with neural regeneration (Gotz and Huttner, 2005). Furthermore, NCs play pivotal roles in brain development and the maintenance of internal environmental stability. Dysfunction of NCs can disrupt the CNS, leading to the development of various diseases. NC-driven neuroimmune responses and whole-brain metabolic diseases are two key components of neurodegenerative disorders (Bernier et al., 2020). Hypoxia, a common condition in cerebrovascular diseases, results in the accumulation of lactate. Recent studies revealed that p53 lysine lactylation mediated proinflammatory phenotypes in microglia under hypoxic conditions, leading to increased expression of proinflammatory cytokines such as iNOS, IL-6, IL-1β, and TNF-α (Fei et al., 2024). Increasing evidence suggests that, in addition to participating in energy metabolism, lactate in the CNS also serves as a donor for protein lactylation (Yang et al., 2024a). Currently, several studies have confirmed that non-histone modifications in NCs can alter their activities, further influencing the progression of diseases.

Interruption of cerebral blood flow leads to hypoxia and insufficient supplies of energy substrates in brain tissue, triggering mitochondrial dysfunction and bioenergetic stress (Li et al., 2024d). Consequently, lactate levels are often elevated in the cerebrospinal fluid of stroke patients (Liang et al., 2021). Increased lactate can inhibit the release of functional mitochondria from astrocytes, thereby exacerbating neuronal damage after cerebral ischemia (Wal et al., 2024). However, low-density lipoprotein receptor-related protein-1 (LRP1) can promote the transfer of healthy mitochondria from astrocytes to neurons by reducing the lactylation of ADP-ribosylation factor 1 (ARF1), thus protecting neurons from ischemic reperfusion injury. Zhou’s research using targeted metabolomics revealed that knocking down LRP1 in astrocytes activated both glycolysis and oxidative phosphorylation processes. Additionally, LRP1 knockdown significantly increased glucose uptake in these cells. ARF1, which was lactylated at K73 site, exhibited high functional similarity to LRP1 and potentially played a role in mitochondrial release. LRP1 regulated lactate metabolism in astrocytes and ARF1 lactylation, influencing mitochondrial extrusion and transfer. Lactylation of ARF1 at K73 site may exacerbate brain injury by limiting mitochondrial transfer, while reducing lactylation at this site could protect neurons by promoting mitochondrial transfer (Zhou et al., 2024a).

Similarly, high-altitude cerebral edema, an end-stage manifestation of acute mountain sickness (Deweber and Scorza, 2010), is closely associated with inflammation (Yu et al., 2022). Hypoxia exacerbates microglial neuroinflammatory responses through the accumulation of lactate. Global profiling of protein lactylation revealed that extensive lactylation occurred after hypoxic induction, and these modifications preferentially targeted protein complexes such as the NuRD complex, ribosome biogenesis complex, spliceosome complex, and DNA replication complex. The lactylation of proteins in microglia further alters their functions and contributes to neuroinflammatory responses under hypoxic conditions, serving as a crucial factor in disease progression (Jiang et al., 2024a).

Neovascularization in the eye is a major cause of blindness (Sulaiman et al., 2016). Retinal microglia are involved in hypoxia-induced angiogenesis and vascular disease (Yin et al., 2017). Wang et al. reported that 77 lactate sites in 67 proteins were significantly upregulated by hypoxia, among which the transcription factor Yin-Yang 1 (YY1) was lactated at K183 (Wang et al., 2023a). In microglia, YY1 could directly bind to the promoter of the classical proangiogenic factor FGF2 and promote its transcription (Baritaki et al., 2009; Gu et al., 2022a), and this process was regulated by the lactate modification level of YY1. In addition, the K183 site lactylation of YY1 was regulated by p300. Under hypoxic conditions, the expression of p300 in HMC3 microglia increased, leading to increased lactate modification by YY1 and increased FGF2 expression, which subsequently facilitated angiogenesis (Wang et al., 2023a).

The alleviation of hypoxic-ischemic encephalopathy (HIE) is significantly facilitated by the polarization of microglia toward the M2 phenotype, a process that exhibits neuroprotective properties (Zhu et al., 2021; Hong et al., 2022). Wang reported that aberrant cyclic GMP-AMP synthase (cGAS) expression in the microglia and umbilical artery blood of HIE patients linked high cGAS lactylation to neuronal injury. Targeting cGAS lactylation has emerged as a novel strategy for treating HIE by promoting microglial M2 polarization and inhibiting glycolysis (Wang et al., 2024c).

### 3.2 Effect of non-histone lactylation on psychiatric disorders

Under pathophysiological conditions, Lactate plays a different role in the occurrence and development of psychiatric disorders (Hylen et al., 2023; Huang et al., 2024c). Moderate exercise in humans results in the generation of lactate, which effectively alleviates anxiety (Carapeto and Aguayo-Mazzucato, 2021), and non-histone lactylation also has a wide range of effects on psychiatric diseases. Exercise-induced lactate significantly enhanced the lactylation of various synaptic proteins, particularly synaptosome-associated protein 91 (SNAP91), a crucial molecule in the endocytosis of synaptic vesicles (AlAbdi et al., 2023). The lactylation of SNAP91 promoted the formation of synaptic structures and neuronal activity in the medial prefrontal cortex, thereby accelerating recovery from chronic restraint stress (CRS). Furthermore, the increased lactylation of SNAP91 through exercise helped prevent anxiety-like behaviors in CRS mice (Yan et al., 2024a).

Alzheimer’s disease (AD) is a progressive neurodegenerative disorder that leads to severe cognitive decline. Excessive ferritinophagy and the consequent release of excess iron ions can induce ferroptosis (Fuhrmann et al., 2020). Ferroptosis has been observed in microglia of patients with AD (Long et al., 2022). The study by An et al. found that the regulation of ferritinophagy was mediated by tau K677 lactylation through the MAPK pathway. Furthermore, mutating K677 to R reduced tau lactylation, inhibited ferroptosis, improved memory, and reduced neuronal damage in an AD mouse model. Notably, these benefits were independent of the phosphorylation level of tau (An et al., 2024). Based on the above, non-histone lactylation can promote functional changes in the corresponding proteins, which can influence the development of brain disease (Table 2).

**TABLE 2:** Non-histone lactylation in brain diseases.

Disease	Site of lactylation	Mechanism	Writer/Eraser	Ref
Ischemic stroke	ARF1 K73	Exacerbated brain injury by limiting mitochondrial transfer	Unknown	Zhou et al. (2024a)
High-altitude cerebral edema	NuRD complex	Increased inflammation response under hypoxic conditions	Unknown	Jiang et al. (2024a)
Ocular neovascularization	YY1 K183	Enhanced FGF2 transcription and promoted angiogenesis	p300	Wang et al. (2023a)
Hypoxic-ischemic encephalopathy	cGAS K162	Promoted the transformation of microglia into M1 type	Unknown	Wang et al. (2024c)
Chronic restraint stress	SNAP91 K885	Promoted the formation of synaptic structures and neuronal activity	Unknown	Yan et al. (2024a)
Alzheimer’s disease	tau K677	Promoted ferroptosis, decreased memory, and increased neuronal damage	Unknown	An et al. (2024)

## 4 Involvement in inflammation and immune response processes

**FIGURE 3:**
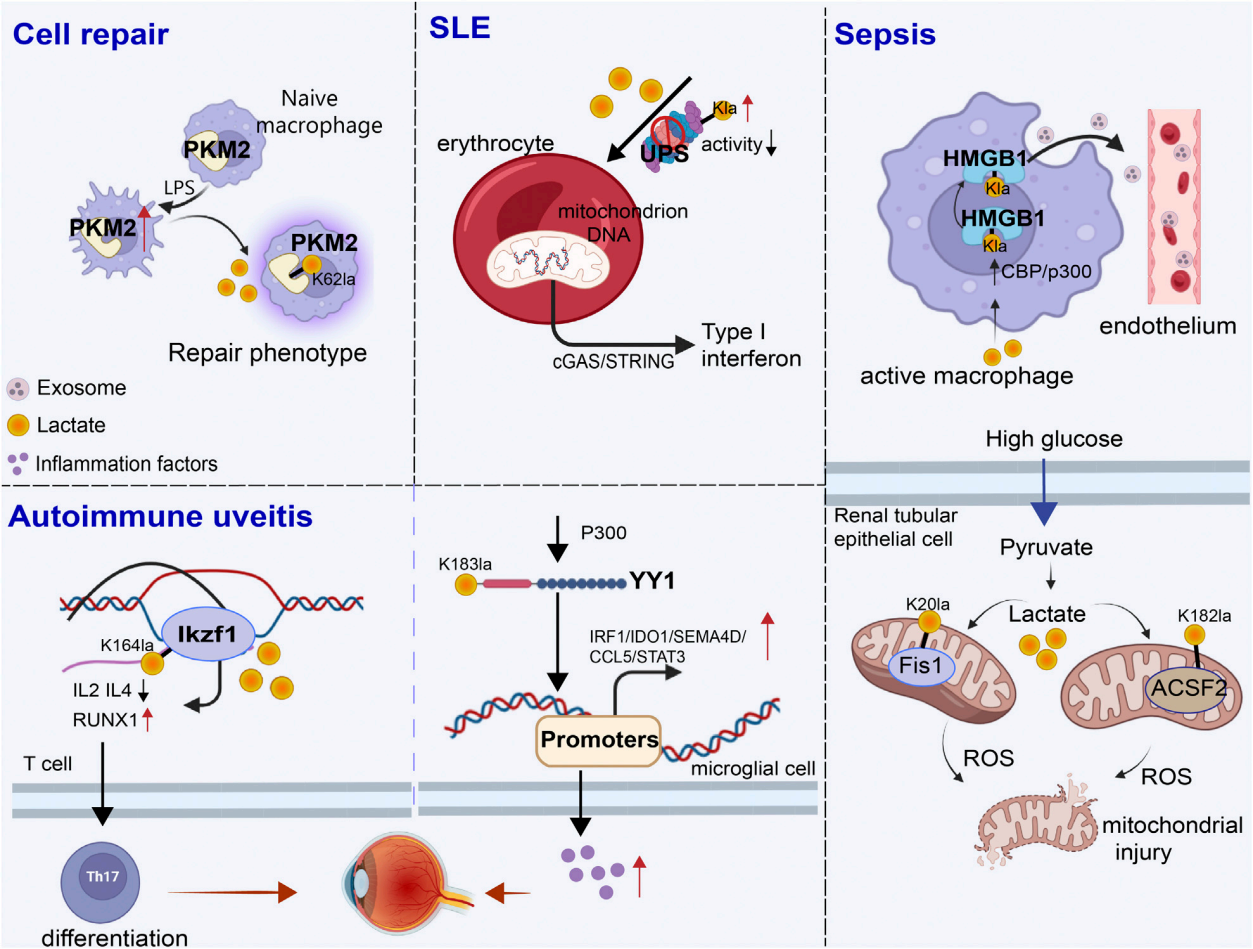
Role of non-histone lactylation in inflammation and immune regulation. The accumulated lactate is involved in non-histone-regulated inflammatory and immune responses, triggering the activation of a series of cell signaling pathways. We presented four types of inflammatory and immune processes involving non-histone lactylation. LPS, lipopolysaccharide; UPS, ubiquitin‒proteasome system; SLE, systemic lupus erythematosus; Kla, lysine lactylation; ROS, reactive oxygen species.

Non-histone lactylation is also associated with inflammation- and immunity-driven pathological processes, influencing their progression through various pathways. By modulating protein activity and cellular metabolism, it plays an important role in coordinating immune responses, tissue repair, and inflammatory reactions (Figure 3) (Table 3). Kla occurs in erythrocytes in systemic lupus erythematosus (SLE) patients and regulates the activation of the ubiquitin‒proteasome system (UPS) via metabolic switches, resulting in the failure of mitochondria in erythrocytes to be cleared via autophagy (Li et al., 2022). Once abnormal red blood cells are taken up by macrophages, the mitochondrial DNA within erythrocytes stimulates a powerful inflammatory pathway called the cGAS/STING pathway, which in turn drives the production of type I interferons, thus causing SLE (Caielli et al., 2021).

**TABLE 3:** Non-histone lactylation in inflammation and immune response processes.

Disease	Site of lactylation	Mechanism	Writer/Eraser	Ref
Systemic lupus erythematosus	UPS	Caused failure of mitochondrial clearance in erythrocytes	Unknown	Caielli et al. (2021)
Sepsis	HMGB1	Disrupted endothelial integrity leading to endothelial barrier dysfunction	p300, CBP	Yang et al. (2022)
Sepsis-induced acute kidney injury	Fis1 K20	Promoted excessive mitochondrial fission, augmenting mitochondrial apoptosis	Unknown	An et al. (2023)
Hyperglycemia	ACSF2 K182	Caused excessive accumulation of ROS, leading to mitochondrial damage	Unknown	Chen et al. (2024d)
Autoimmune uveitis	Ikzf1 K164	Regulated the transcription and expression of genes associated with Th17 differentiation	Unknown	Fan et al. (2023b)
Autoimmune uveitis	YY1 K183	Upregulated inflammatory cytokine secretion	p300	Huang et al. (2024a)
Wound healing	PKM2 K62	Increased the repair-phenotype macrophages, and decreased the proinflammatory-phenotype macrophages	Unknown	Wang et al. (2022)

Sepsis is a life-threatening disease characterized by organ dysfunction and a dysregulated inflammatory response (Retzlaff et al., 2017; Winkler et al., 2017). Lactate significantly affects the functional capacity of immune cells in sepsis and septic shock (Zhang et al., 2024a). Recent investigations have shown that the amount of lactate in the blood of patients with sepsis is proportional to the amount of HMGB1 (Yu et al., 2024b). During multimicrobial sepsis, macrophages are capable of absorbing extracellular lactate through MCTs. HMGB1 is released by activated macrophages to coordinate the inflammatory response. Yang et al. found that lactate could be taken up, leading to HMGB1 lactylation in a p300/CBP-dependent mechanism. Furthermore, lactate promoted HMGB1 acetylation by inhibiting the SIRT1 while simultaneously recruiting the p300/CBP (Shang et al., 2022; Sun et al., 2023d) into the nucleus through the mediation of G protein-coupled receptor 81 (GPR81). Lactylated/acetylated HMGB1 in turn translocated into lysosomes of macrophages, which was then released via the exosomal secretion pathway. Consequently, this disrupted endothelial integrity and increased vascular permeability, leading to endothelial barrier dysfunction and ultimately promoting the development of sepsis. Importantly, reducing lactate production and/or inhibiting GPR81-mediated signaling *in vivo* has been found to decrease HMGB1 levels and improve survival outcomes in patients with multimicrobiome sepsis (Yang et al., 2022). Meanwhile, lactate may serve as an independent risk factor for sepsis-induced acute kidney injury (SAKI) (Liu et al., 2019; An et al., 2023). Fission 1 (Fis1) is a mitochondrial outer membrane adapter protein that interacts with dynamin-related protein 1 (DRP1) to mediate mitochondrial fission (Bam et al., 2021). Lactate mediated the lactylation of mitochondrial Fis1, specifically at the K20 residue. The increase in Fis1 K20la promoted excessive mitochondrial fission, subsequently inducing ATP depletion, augmenting mitochondrial apoptosis, and accelerating the progression of SAKI. Conversely, reducing lactate production and Fis1 lactylation improved renal tubular epithelial cell damage and alleviated SAKI (An et al., 2023). In addition, changes in blood glucose levels are particularly significant in sepsis patients, as sepsis often induces stress hyperglycemia (Wang et al., 2021b). Lactylation of Acyl-CoA Synthetase Family Member 2 (ACSF2) caused excessive accumulation of reactive oxygen species (ROS), leading to mitochondrial damage in HK-2 cells stimulated by high glucose treatment (Chen et al., 2024d).

Autoimmune uveitis, a prevalent form of immune-mediated blindness, involves aberrant T helper 17 (Th17) cell differentiation, which relies heavily on glycolytic metabolism. The level of lactylation in CD4^+^ T cells increased as experimental autoimmune uveitis (EAU) progresses, and this lactylation played a pivotal role in regulating Th17 cell differentiation. Inhibition of lactylation not only suppressed Th17 differentiation but also alleviated EAU-induced inflammation. Ikzf1, a member of the Ikaros transcription factor family, is crucial in regulating lymphocyte development (Vesely et al., 2017). Notably, Ikzf1 is indispensable for Th17 differentiation, as naïve CD4^+^ T cells lacking Ikzf1 are unable to differentiate into Th17 cells (Bernardi et al., 2021). Lactate modification at Ikzf1’s K164 site influenced Th17 differentiation via the modulation of IL2, IL4, Tlr4, Runx1 and other genes (Fan et al., 2023b). Furthermore, activated microglia in the retina are vital to the progression of autoimmune uveitis (Cai et al., 2022). YY1, which was also involved in various inflammatory and immune-mediated diseases, underwent increased lactylation in retinal microglia in the EAU group. This lactylation of YY1 promoted the activation of microglia, increasing their proliferation and migration capabilities, which led to microglial dysfunction that contributed to uveitis. Additionally, the inhibition of p300 reduced YY1 lactylation and suppressed microglial inflammation (Huang et al., 2024a).

During the wound healing process, macrophage phenotypes changed dynamically over time, characterized by a decrease in the expression of proinflammatory genes and an increase in the expression of tissue repair genes (Kusnadi et al., 2019). These changes have promoted wound healing. Pyruvate kinase M2 (PKM2), as one of the substrates of lactylation, is upregulated in bone marrow-derived macrophages (BMDMs) upon lipopolysaccharide (LPS) stimulation. After lactylation, PKM2 increased the expression of Arg1, a marker of repair-phenotype macrophages, and decreased the expression of iNOS, a marker of proinflammatory-phenotype macrophages (Han et al., 2023), indicating that lactate promoted the transformation of macrophages to a repair phenotype by activating PKM2 (Wang et al., 2022). However, PKM2 serves as a prominent isoform of pyruvate kinase in endothelial cells and plays a crucial role in endothelial cell proliferation (Ziogas et al., 2021). Excessive activation of PKM2 triggered lactate accumulation, disrupting glucose metabolism. The glycolysis–lactate pathway was correlated with Twist1 and p300/CBP, leading to the lactylation of Twist1. This lactylation subsequently resulted in the phosphorylation and nuclear translocation of Twist1. The activation of the TGF-β/Smad2 pathway promoted the progression of EndoMT, thereby inducing a fibrotic phenotype (Xu et al., 2024).

## 5 Oncogenesis and progression

### 5.1 Tumorigenesis

As epigenetic regulators, RNA modifications, among which N6-methyladenosine (M6A) is the most abundant RNA modification in eukaryotic cells (Deng et al., 2018), have received increasing attention in recent years. Methyltransferase 16 (METTL16) is primarily functions in cells by participating in the N6-adenosine methylation of RNA. This modification has profound impacts on RNA splicing, translation, and stability, thereby regulating gene expression (Su et al., 2022). Furthermore, METTL16 has shown tumorigenesis and tumor-promoting capabilities in a M6A-dependent manner in a variety of tumors (Wang et al., 2021c; Dai et al., 2022b). High copper content promoted the lactylation of METTL16 at the K229 site by enhancing the interaction between AARS1 and AARS2 with METTL16, ultimately leading to cuproptosis. Disruptions in copper homeostasis are associated with various types of tumors. The role of copper in modulating the lactylation of non-histone METTL16 provides a novel direction for the prevention and treatment of tumors (Sun et al., 2023a).

Discoidin, CUB, and LCCL domain-containing protein 1 (DCBLD1) have been identified as oncogenes involved in multiple regulatory mechanisms of tumor progression (Wang et al., 2020). Lactate not only increased DCBLD1 expression but also induced the lactylation of DCBLD1 at K172 site, thereby stabilizing DCBLD1 expression. This upregulation enhanced the expression and enzyme activity of glucose-6-phosphate dehydrogenase (G6PD), which stimulated the pentose phosphate pathway (PPP), promoting the proliferation and metastasis of cervical cancer cells (Meng et al., 2024a).

ATP metabolism is crucial for cellular processes, with adenylate kinase 2 (AK2) being a key enzyme that catalyzes the transfer of phosphate between ATP and AMP to form ADP (Cheng et al., 2023). Yang’s team reported that AK2 lactylation was linked to poor HCC prognosis, promoted cell proliferation and oncogenic pathways, and suppressed liver-specific and p53 pathways. Mass spectrometry confirmed that AK2 was lactated in the ATP-binding region. In HepG2 and Hep3B cells, lactate or excess glucose increased AK2 lactylation without affecting acetylation. Elevated AK2 lactylation reduced AK2 activity and promoted energy dysregulation, proliferation, and migration in HCC cells (Yang et al., 2023b). Additionally, CCNE2, as mentioned above, is a pivotal cell cycle protein and has been implicated in abnormal activities in numerous tumors, influencing tumor proliferation and oncogenesis. The overexpression of CCNE2 also contributes to tumor drug resistance (Chu et al., 2021). The lactylation of CCNE2 promoted the proliferation, migration, and invasion of HCC cells. On the other hand, SIRT3 regulated the Kla level on CCNE2, which in turn modulated the cell cycle, ultimately leading to an impediment to HCC progression (Jin et al., 2023).

Intrahepatic cholangiocarcinoma (iCCA) is a fatal malignant tumor of the biliary system (Tiemin et al., 2020). NCL, the most abundant RNA-binding protein found in the nucleolus, underwent lactylation at K477 site by the acyltransferase p300 under conditions of hyperactive glycolysis. This lactylation further promoted the proliferation and invasion of iCCA cells. NCL also upregulated the expression level of MAPK-activated death domain protein (MADD). NCL K477la promoted MADD expression through RNA splicing, thereby preventing premature termination of translation. NCL lactylation, MADD translation and subsequent ERK activation promoted xenograft tumor growth and were associated with overall survival in iCCA patients (Yang et al., 2024b).

Methylcytosine (m5C), the product of RNA methylation, plays a crucial role in RNA metabolic regulation and various tumorigenic processes (Wang et al., 2023c). The study by Chen et al. found that NSUN2, an m5C methyltransferase, induces metabolic reprogramming that enhances glucose metabolism in colorectal cancer cells, leading to increased lactate production. Specifically, Lactate not only activated the transcription of NSUN2 through the lactylation of H3K18 but also induced the lactylation of NSUN2 at the K356 site. This lactylation facilitated the capture of target RNAs and the m5C modification of ENO1 mRNA. Overall, this positive feedback loop involving NSUN2, YBX1, and m5C-ENO1 further upregulated glycolysis and promoted the occurrence and development of colorectal cancer (Chen et al., 2024a).

Tumor cell stemness influences tumor growth, drug resistance, metastasis and recurrence, and interacts with the tumor microenvironment, making it a key factor in tumor malignancy progression (Ding et al., 2015; Juric et al., 2021). Liver cancer stem cells (LCSCs) constitute a crucial subpopulation within heterogeneous HCC tissues and are considered the fundamental cause of liver cancer initiation, progression and deterioration (Wang et al., 2019). Researchers have used transcriptomics, metabolomics, and Seahorse XF analysis to compare glycolysis levels between HCC cells and LCSCs (Feng et al., 2024). LCSCs exhibited significantly higher mRNA and protein expression levels of glycolysis-related enzymes compared to HCC cells, resulting in an increased glycolytic rate and elevated intracellular lactate content. Consequently, the overall level of lactylation in LCSCs was significantly greater. Furthermore, the level of Kla on non-histone proteins in liver LCSCs was significantly elevated, particularly the lactylation of ALDOA at K230/K322 site. Lactylation of ALDOA at these sites caused the dissociation of ALDOA from DDX17. Upon the nuclear translocation of DDX17, its nuclear expression enhanced its tumorigenicity and stem-like characteristics by facilitating the binding of SOX2 to its target genes (Alqahtani et al., 2016). Additionally, as the tumor grows, the tumor microenvironment becomes hypoxic. Hypoxia triggered lactylation of the serine hydroxymethyl transferase 2 (SHMT2) protein and enhanced its stability, thereby promoting glycolysis and stemness in esophageal carcinoma cells (Qiao et al., 2024). Extensive evidence pointed to glioma stem cells as key drivers of progression and recurrence, with a unique metabolic profile marked by increased glycolysis. PTBP1 lactylation inhibited its proteasomal degradation by reducing its interaction with TRIM21. This modification enhanced PTBP1’s RNA-binding ability, stabilizing PFKFB4 mRNA and promoting glycolysis (Zhou et al., 2024b). Based on the above, non-histone lactylation plays an important role in tumor growth (Table 4). Therefore, targeting specific sites involved in the regulation of Kla may be an effective cancer treatment strategy.

**TABLE 4:** Non-histone lactylation in tumorigenesis.

Disease	Site of lactylation	Mechanism	Writer/Eraser	Ref
Gastric cancer	METTL16 K229	Caused cuproptosis	AARS1, AARS2/SIRT2	Sun et al. (2023a)
Cervical cancer	DCBLD1 K172	Enhanced the expression and enzyme activity of G6PD	Unknown	Meng et al. (2024a)
Hepatocellular carcinoma	AK2 K28	Promoted energy dysregulation	Unknown	Yang et al. (2023b)
Hepatocellular carcinoma	CCNE2 K347, K348	Modulated the cell cycle	SIRT3	Jin et al. (2023)
Intrahepatic cholangiocarcinoma	NCL K477	Promoted MADD expression by RNA splicing, thereby prevented premature termination of translation, further activated ERK	p300	Yang et al. (2024b)
Colorectal cancer	NSUN2 K356	Upregulated glycolysis	Unknown	Chen et al. (2024a)
Hepatocellular carcinoma	ALDOA K230, K322	Enhanced tumorigenicity and stem-like characteristics	Unknown	Feng et al. (2024)
Esophageal cancer	SHMT2	Enhanced SHMT2 stability, thereby led to increased MTHFD1L expression	Unknown	Qiao et al. (2024)
Glioma	PTBP1 K436	Promoted glioma progression and glioma stem cells maintenance	SIRT1	Zhou et al. (2024b)

### 5.2 Tumor microenvironment

The large amount of lactate produced by glycolysis in the tumor microenvironment alters it as well (Pourbaghi et al., 2023; Zhang et al., 2023b). Regulatory T cells (Tregs) play a critical role in maintaining the immunosuppressive tumor microenvironment. Studies have shown that lactate improves the stability and function of Treg cells, while lactate degradation reduces the induction of Treg cells, enhances antitumor immunity, and reduces tumor growth (Watson et al., 2021; Lopez Krol et al., 2022; Sun et al., 2023b). Lactate regulated the generation of Treg cells by lactylating MOESIN at K72 site and strengthened the interaction between MOESIN and TGF-βRI through the provision of an additional hydrogen bond. Anti-PD1 therapy has been extensively utilized in the treatment of tumors, yet the phenomenon of therapeutic resistance in tumors remains a significant concern. Notably, the combination of a lactate dehydrogenase inhibitor and an anti-PD-1 antibody could enhance antitumor efficacy, suggesting a novel direction for improving treatment outcomes (Gu et al., 2022b). Chen’s study revealed that, compared with that in adjacent normal tissues, apolipoprotein C2 (APOC2) protein expression was upregulated in tumors. Lactate-induced extracellular free fatty acid (FFA) release was shown to be dependent on APOC2, and its lactylation site was detected as K70 site. Lactate enhanced APOC2-K70la, promoting FFA release and Tregs. APOC2-K70R reduced Tregs, increased CD8^+^ T cells, and decreased Treg activity (Chen et al., 2024e). Moreover, lactylation of RIG-I inhibited the recruitment of NF-κB to the Nlrp3 promoter in macrophages, leading to reduced Nlrp3 transcription. This decrease in Nlrp3 affected the immunosuppressive function of Tregs and the antitumor activity of CD8^+^ T cells (Gu et al., 2024).

### 5.3 DNA damage response

**FIGURE 4:**
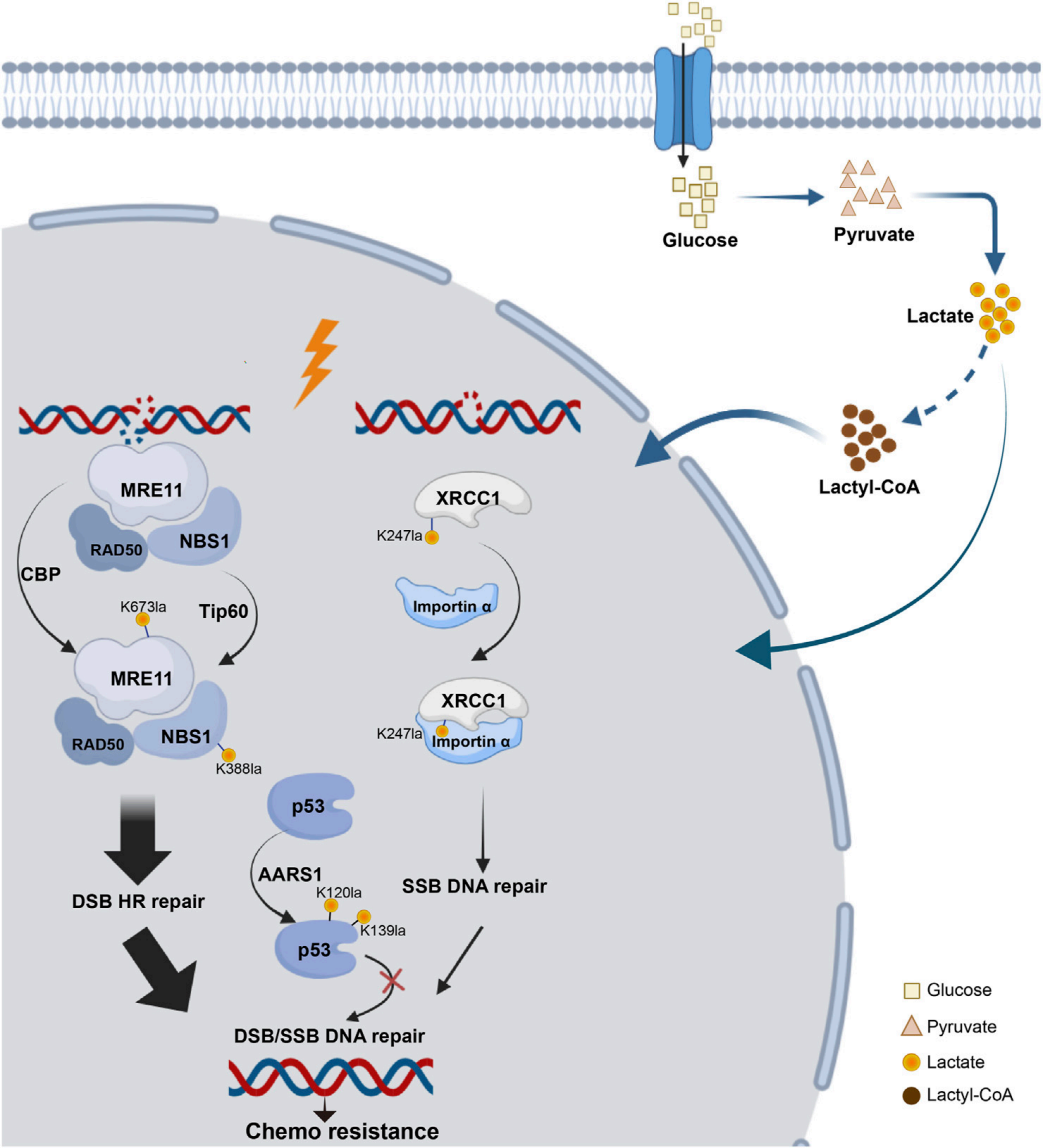
Lactylation network of DNA damage repair proteins. The process of DNA damage repair following DSB or SSB involves key components essential for HR repair, such as MRE11 and NBS, as well as critical proteins involved in base excision repair, such as XRCC1 and tumor suppressors like p53. All of these proteins have been found to undergo lactylation, which can influence chemotherapy resistance.

DNA damage profoundly affects genomic stability, especially in the form of double-strand breaks (DSBs), which are linked to various diseases, such as cancer, neurological disorders, growth retardation, and immunodeficiency (Birkbak et al., 2012; Pilger et al., 2021; Wang et al., 2021a). Recent studies have revealed that the repair of DSBs via HR is regulated by the Warburg effect, with lactate promoting tumor cell resistance to DNA-damaging agents through lactylation. Some studies have confirmed that lactylation of essential proteins is linked to DNA damage repair (Figure 4).

Overactivation of HR caused resistance to cancer platinum-based neoadjuvant chemotherapy (NAC). MRE11, a key MRN complex component (Horak et al., 2022), repairs DSBs. Researchers identified MRE11 with K673 as its primary lactylation site. The inhibition of MCTs, which elevated cellular lactate levels, enhanced MRE11 K673 site lactylation. CBP acetyltransferase mediated this lactylation, whereas SIRT1 and SIRT2 reversed it (Chen et al., 2024g). Using 4D lactylation proteomics and the STRING database, researchers confirmed the role of NBS1 in DNA damage repair. Chen’s study revealed lactate-induced lactylation of NBS1 at K388 site. Elevated levels of lactate dehydrogenase A (LDHA) and NBS1 K388 lactylation observed in NAC-resistant tumors were strongly associated with clinical resistance to NAC and indicated a poor prognosis for patients. These findings suggest potential targets for overcoming chemotherapy resistance (Chen et al., 2024b).

Additionally, lactate accumulation upregulated XRCC1 lactylation at K247 site, enhancing DNA repair in glioblastoma and promoting tumor cell resistance to radiotherapy and chemotherapy. Mechanically, high ALDH1A3 expression activated the glycolytic pathway through interaction with PKM2, increasing lactate metabolism in tumor cells. Lactylation of XRCC1 altered its surface charge, increasing its affinity for importin α and leading to nuclear ectopia, which further promoted DNA repair and tumor progression (Li et al., 2024b).

P53 lactylation via the NF-κB/STAT3/SLC4A4 axis not only contributes to the development of resistance to androgen receptor signaling inhibitors and the progression of cancer but also plays a role in DNA damage repair (Chen et al., 2024c). Tumor-derived lactate inhibited p53, a key regulator of DNA damage repair and cell death pathways. They identified AARS1 as a lactate sensor and transferase that catalyzed p53 lactylation at K120 site and K139 site. In a breast cancer mouse model, lactate injection suppressed p53 activity and increased the tumor burden, whereas LDHA knockout had the opposite effects. They reported that high levels of p53 lactylation, facilitated by AARS1 and the lactate-AMP intermediate, led to decreased p53 activity (Zong et al., 2024).

## 6 Understanding non-histone lactylation on the basis of viral insights

In the context of viral infection, non-histone lactylation is an emerging research area, particularly in elucidating how viruses interact with the host. The predominant HPV type detected in cervical cancer is HPV16, and the persistent expression of its E6 and E7 viral oncogenes, along with those of other high-risk HPV types, plays a crucial role in driving the development of cervical cancer (Liu et al., 2018). Expression of HPV16 E6 and E7 promoted cervical cancer cell proliferation by activating the PPP. Mechanistically, HPV16 E6 inhibited G6PD K45 site lactylation to increase G6PD enzyme activity, leading to elevated PPP activity, which is commonly overactivated in tumor cells (Meng et al., 2024b). Emerging studies have demonstrated that viral infection is linked to aberrant RNA N4-acetylcytidine (ac4C) modification, which is dependent on N-acetyltransferase 10 (NAT10) (Hao et al., 2022). The acetyltransferase NAT10 was lactylated at K290 site by α-tubulin acetyltransferase 1 (ATAT1), which induced ac4C modification to promote reactivation of Kaposi’s sarcoma-associated herpesvirus from latency and activated the inflammasome (Yan et al., 2024b). Severe fever with thrombocytopenia syndrome virus (SFTSV), an emerging pathogen with a high fatality rate, is an enveloped, tripartite, segmented, single-stranded negative-sense RNA virus (Li et al., 2021). The YT521-B homology domain family protein YTHDF1 bond to m6A-modified sites on SFTSV RNA, reducing its stability and lowering SFTSV protein translation efficiency. However, the SFTSV virulence factor NSs enhanced YTHDF1 lactylation and drove its degradation, thereby promoting SFTSV replication (Liu et al., 2024).

## 7 Lactylation of other non-histone proteins under pathological and physiological conditions

Autophagy and glycolysis are highly conserved biological processes, with lactate serving as a bridge linking the two through lactylation (Cheng et al., 2019; Sun et al., 2023c). Lactate modifies vacuolar protein sorting 34 (Vps34), activating its kinase activity and promoting autophagy and endolysosomal degradation. Lactate, mediated by TIP60, modified Vps34 at K356 site and K781 site, enhancing its interaction with Beclin1, Atg14L, and UVRAG, thereby promoting autophagy flux and endolysosomal transport (Jia et al., 2023). Additionally, the transcription factor EB (TFEB) plays a key role in regulating lysosomal biogenesis and autophagy (Bo et al., 2023). Lactylation of TFEB at K91 site blocked the interaction between TFEB and the E3 ubiquitin ligase WWP2, preventing TFEB ubiquitination and proteasomal degradation. These factors led to enhanced TFEB activity and increased autophagic flux (Huang et al., 2024b). Neuronal apoptosis plays an important role in traumatic brain injury. Lactylation of mitochondrial Tu translation elongation factor (Tufm) at K286 site inhibited the interaction between Tufm and Tomm40 on mitochondria. This modification impeded the mitochondrial localization of Tufm, leading to the suppression of Tufm-mediated mitophagy and an increase in mitochondria-induced neuronal apoptosis (Weng et al., 2024).

Furthermore, lactylation of runt-related transcription factor 2 (RUNX2) stabilized the protein, thereby promoting osteogenic differentiation in periodontal ligament stem cells (PDLSCs), which has significant implications for periodontal tissue regeneration and bone defect repair (Wu and Gong, 2024).

## 8 Potential therapeutic strategies targeting non-histone protein lactylation

On the basis of the effects of non-histone protein lactylation on diseases, in numerous studies, researchers have explored the therapeutic potential of inhibiting non-histone lactylation by screening small molecule compound libraries or using lactylation transferase inhibitors (Table 5). Li et al. identified a small-molecule compound through high-throughput screening, D34-919, which specifically blocked the interaction between ALDH1A3 and PKM2, effectively overcoming resistance to temozolomide and radiotherapy (Li et al., 2024b). Zong et al. demonstrated the role of p53 lactylation and introduced a novel therapeutic strategy that used β-alanine to inhibit p53 lactylation, thereby restoring its tumor-suppressive activity (Zong et al., 2024). Chen et al. developed an anti-APOC2^K70−Lac^ antibody that sensitized cells to anti-PD-1 therapy *in vivo* (Chen et al., 2024e). Sun et al. reported that combining elesclomol with AGK2, a SIRT2-specific inhibitor, induced cuproptosis in gastric tumors (Sun et al., 2023a). Chen et al. reported that a cell-penetrating peptide that specifically blocked MRE11 lactylation could inhibit HR and sensitize cancer cells to cisplatin and PARP inhibitors (Chen et al., 2024g). An et al. demonstrated that GSK2837808, a lactate dehydrogenase inhibitor that decreased lactate production, attenuated the 3-TYP-induced elevation in Fis1 K20la levels. Chen et al. reported that the antiepileptic drug stiripentol could inhibit NBS1 K388 lactylation, rendering resistant cancer sensitive to DNA-damaging treatment (Chen et al., 2024b).

**TABLE 5:** Potential therapeutic strategies targeting non-histone protein lactylation.

Name	Category	Effectiveness	Mechanisms	Ref
D34-919	PKM2 allosteric activation site inhibitors	Conquered resistance to temozolomide and radiotherapy	Blocked the Interaction between ALDH1A3 and PKM2	Li et al. (2024b)
β-alanine	Non-essential amino acid	Inhibited tumor progression	Disrupted lactate binding to AARS1, reduced p53 lacylation	Zong et al. (2024)
anti-APOC2^K70−lac^ antibody	APOC2 lactylation antibody	Sensitized immunotherapeutic responses	Neutralized extracellular APOC2	Chen et al. (2024e)
AGK2	SIRT2-specific inhibitor	Boosted the therapeutic effectiveness of the copper ionophore-elesclomol	Promoted METTL16 lactylation, inducing cuproptosis	Sun et al. (2023a)
CPP	Cell-penetrating peptide	Sensitized cancer cells to cisplatin and PARPi	Blocked MRE11 lactylation, inhibiting HR	Chen et al. (2024g)
Stiripentol	Lactate dehydrogenase A inhibitor	Sensitized immunotherapeutic responses	Inhibited NBS1 K388 lactylation, decreasing DNA repair efficacy	Chen et al. (2024b)
GSK2837808A	Lactate dehydrogenase inhibitor	Attenuated SAKI	Inhibited Fis1 lactylation, reducing mitochondrial apoptosis	An et al. (2023)
Artemisinin	Antimalarial drug	Suppressed synovial hyperplasia	Interacted with PKM2, allosterically enhancing its lactylation	Wang et al. (2024d)
Dexmedetomidine	α2-adrenergic receptor agonist	Reduced myocardial infarction area and improved contractility	Reduced MDH2 lactylation, alleviating myocardial injury	She et al. (2024)
poly-L-lactic acid	Bioabsorbable material	Promoted skin rejuvenation	Released lactate, enhanced LTBP1 lactylation, stimulating endogenous collagen synthesis	Zou et al. (2024)
Scopolamine	Anticholinergic drugs	Regenerated periodontal tissues and repaired bone defects	Enhanced RUNX2 lactylation, promoting osteogenic differentiation	Wu and Gong (2024)

In addition, fibroblast-like synoviocytes play an important role in rheumatoid arthritis (RA), and the protein p300 targets PKM2, promoting its lactylation, which inhibited RA-FLS proliferation by preventing the translocation of PKM2 to the nucleus, and the antimalarial drug artemisinin could enhance this process (Wang et al., 2024d). She et al. reported that dexmedetomidine (Dex) conferred cardioprotection by reducing the lactylation of malate dehydrogenase 2 (MDH2), thus alleviating myocardial injury (She et al., 2024). Zou et al. discovered that poly-L-lactic acid (PLLA), a bioabsorbable material, could be utilized to promote skin rejuvenation. It continuously released lactate, which entered fibroblasts through MCT1, upregulated the expression of the histone acetyltransferase KAT8, and promoted the lactylation of fibroblasts, thereby inducing the synthesis of type I and type III collagen in fibroblasts (Zou et al., 2024). Notably, scopolamine (SCO), which acts as a modulator of neuronal cell injury, increased the viability of PDLSCs and induced their osteogenic differentiation. Mechanistically, SCO facilitated the lactylation of RUNX2, with lactylation at the K176 residue increasing RUNX2 protein stability via deubiquitination (Wu and Gong, 2024).

In summary, lactate, which was once regarded merely as a metabolic byproduct, is now understood to be the inevitable final product of glycolysis and plays numerous regulatory roles in various types of cells. Non-histone lactylation, a novel posttranslational modification, influences various cellular processes, including metabolism, gene expression, signal transduction, immune regulation, and particularly the M1/M2 polarization of macrophages. It plays a critical role in the development and progression of cardiovascular diseases, neurological disorders, autoimmune diseases, and other conditions. Depending on the pathological characteristics of each disease, non-histone lactylation has complex effects, either by promoting or inhibiting disease progression. Notably, owing to the Warburg effect, non-histone lactylation plays a pivotal role in directly regulating oncogene expression, influencing tumor growth, invasion and metastasis. Furthermore, it can induce the differentiation of Treg cells and promote DNA damage repair, thereby leading to tumor immune evasion and resistance to chemotherapy drugs. Additionally, it can impact disease pathology by modulating virus‒host interactions. The distinctiveness and extensive biological effects of this modification make it a promising area for exploring cellular adaptive responses, inflammatory reactions, and tumor progression. In the future, further investigations into the molecular mechanisms and disease-specific roles of non-histone lactylation will likely provide new targets and strategies for disease diagnosis and treatment.
