# Correction: Non-histone lactylation: unveiling its functional significance

**DOI:** 10.3389/fcell.2025.1707124

**Published:** 2025-09-30

**Authors:** Pusong Shi, Yongjie Ma, Shaolu Zhang

**Affiliations:** ^1^ School of Integrative Medicine, Tianjin University of Traditional Chinese Medicine, Tianjin, China; ^2^ Laboratory of Cancer Cell Biology, Tianjin Medical University Cancer Institute and Hospital, National Clinical Research Center for Cancer, Tianjin, China; ^3^ Tianjin’s Clinical Research Center for Cancer, Tianjin, China; ^4^ Key Laboratory of Breast Cancer Prevention and Therapy, Ministry of Education, Tianjin Medical University, Tianjin, China; ^5^ Key Laboratory of Cancer Prevention and Therapy, Tianjin, China; ^6^ State Key Laboratory of Druggability Evaluation and Systematic Translational Medicine, Tianjin, China

**Keywords:** non-histone, lactylation, posttranslational modification, lysine, metabolism

The funder Tianjin Science and Technology Major Project (22JCZDJC00610 to YM) was erroneously omitted.

The original version of this article has been updated.

